# Pharmacogenomics, human genetic diversity and the incorporation and rejection of color/race in Brazil

**DOI:** 10.1057/biosoc.2014.21

**Published:** 2015-03-01

**Authors:** Ricardo Ventura Santos, Gláucia Oliveira da Silva, Sahra Gibbon

**Affiliations:** a Escola Nacional de Saúde Pública/ FIOCRUZ & Museu Nacional/UFRJ, Rio de Janeiro, Brazil; b Universidade Federal Fluminense, Niterói, Brazil. glaucia.o.silva@gmail.com; c University College, London, UK. s.gibbon@ucl.ac.uk

**Keywords:** color/race, race mixture, anthropology of science, pharmacogenomics, health policy, Brazil

## Abstract

Public funding for research on the action of drugs in countries like the United States requires that racial classification of research subjects should be considered when defining the composition of the samples as well as in data analysis, sometimes resulting in interpretations that Whites and Blacks differ in their pharmacogenetic profiles. In Brazil, pharmacogenomic results have led to very different interpretations when compared with those obtained in the United States. This is explained as deriving from the genomic heterogeneity of the Brazilian population. This article argues that in the evolving field of pharmacogenomics research in Brazil there is simultaneously both an incorporation and rejection of the US informed race-genes paradigm. We suggest that this must be understood in relation to continuities with national and transnational history of genetic research in Brazil, a differently situated politics of Brazilian public health and the ongoing valorization of miscegenation or race mixture by Brazilian geneticists as a resource for transnational genetic research. Our data derive from anthropological investigation conducted in INCA (Brazilian National Cancer Institute), in Rio de Janeiro, with a focus on the drug warfarin. The criticism of Brazilian scientists regarding the uses of racial categorization includes a revision of mathematical algorithms for drug dosage widely used in clinical procedures around the world. Our analysis reveals how the incorporation of ideas of racial purity and admixture, as it relates to the efficacy of drugs, touches on issues related to the possibility of application of pharmaceutical technologies on a global scale.

## Introduction

In the same way that they access their bank accounts and e-mail, physicians and other health professionals in any part of the world can download to their computers, tablets or cell phones a program that calculates the dosage of warfarin anticoagulant to administer to their patients. Since the 1950s warfarin has been the anticoagulant most used worldwide and it is routinely used whenever patients require medical treatment that involves an anticoagulant. One of the most widely used algorithms is available on the website www.warfarindosing.org, from Washington University at St. Louis, USA, which had been visited more than 580 000 times as of April 2014. The algorithm was developed by “international collaborations of biostatisticians, geneticists, pharmacists, and physicians who share anonymous data to improve warfarin dosing”.

Even those who do not routinely treat hospital patients can easily use the site. A click on the option “warfarin dosing” opens a page entitled “Required patient information”. The page has a number of spaces to be filled in with information, including the patient’s age, sex, weight, height, whether a smoker, as well as previous clinical history, other medications taken and seven genetic characteristics. Of particular relevance to the topic of this article are two specific questions, one regarding the “ethnicity” of the patient (with the options “non-Hispanic”, “Hispanic” and “unknown”) and the other regarding “race” (with the options “African American or Black”, “American Indian or Alaska Native”, “Asian or Indian Subcontinent”, “Native Hawaiian or other Pacific Islander”, “White, Caucasian, or Middle Eastern” and “Other”).

Pharmacogenetics is a branch of medical science specializing in the study of how genetic characteristics affect responses to medications. A fundamental premise of pharmacogenetics is that the efficacy and safety of the use of a drug to treat patients may vary, depending on the genetic profile of the individuals and of populations. Two important pioneers in the field have been the North American Arno Motulsky (see Motulsky, 1964; Gurwitz and Motulsky, 2007) and the German–Canadian doctor Werner Kalow (Jones, 2013). In 1962, Kalow published a book entitled *Pharmacogenetics: Heredity and Response to Drugs* that has become an important point of reference for researchers in this area (Kalow, 1962). More recently, Kalow, with other researchers, published *Pharmacogenomics, Second Expanded Edition* that updated and amplified the previous work (see Kalow *et al*, 2005). While the new book maintained a focus on genes (or polymorphisms) that determine differences in the metabolism of certain drugs, it added an approach based on DNA. As a consequence, pharmacogenetics also came to be called pharmacogenomics (see Suarez-Kurtz, 2010).

In a provocative analysis, the anthropologist Fullwiley (2007) challenges what she calls “institutionalizing race at the molecular level” in the research practices of pharmacogenomics in the United States. She argues that with the increasing emphasis on the application of biomedical technology at the level of the individual (“individualized medicine”), as in the case of pharmacogenomics, a revival of racialized approaches has occurred. The algorithm to calculate the dosage of warfarin may be taken as an example of this “reinscription of race” to which Fullwiley refers. The irony of this development has not been lost to anthropologists and science, technology and society (STS) scholars, since it was from genetics, especially in the second half of the twentieth century, that a powerful denial of the biological concept of race emerged. Scholars like Fullwiley (2007), Bliss (2011, 2012), Lee (2005, 2012) and Montoya (2007, 2011) argue that the “molecularizing of race” in pharmacogenomics in the United States is a dynamic concept with multiple and complex aspects. This not only determines how scientists look at biological differences between human populations in socio-cultural terms, but may have many further effects including, among others, how inequalities in health are related to ethnic/racial categories, and how agencies that regulate and finance research are influenced to include questions relating to race. Lee refers to the broader institutional context in which these developments are taking place in the United States in terms of an “infrastructure for racialization”. She sees these developments in terms of a growing alignment between “research on human genetic variation that maps genes to social categories of race[…] the pervasive use of race as a proxy for risk in clinical medicine, and the search for new ‘racially inscribed’ market niches by the pharmaceutical industry” (Lee, 2005, p. 2133).

The criticisms voiced by Fullwiley, Bliss, Montoya and Lee are part of broader views about the perspective on race that has emerged in the context of new genetic technologies (see Rose, 2007; Koenig *et al*, 2008; Whitmarsh and Jones, 2010; Kahn, 2013). Recent analyses have discussed both the ‘promise’ and the ‘dangers’ involved in the revival of the idea of race, as well as the ways it has been applied in fields related to health regulation, knowledge and care. As the editors of the book *Revisiting Race in a Genomic Age* commented, “contrary to expectation and hopes, post-genomic science has revived the notion of racial category as an indicator of biological difference” (Koenig *et al*, 2008, p. xx). The revived notion of race and the controversies that surround it exemplify how old and recent categories, practices and institutions have acquired new emphases and significance in the context of the emergence of the new genetic technologies, including pharmacogenomics.

Exemplified by the authors cited above, most criticism of the racialization of biomedical technologies and practices comes from the social sciences, especially anthropology and sociology, as well as the field of STS.^1^ In Brazil, in what can be regarded as an internal national critique (that nevertheless aims to inform global research agendas), influential researchers in the field of pharmacogenomics have questioned the use of ethno/racial categories as measures of biological variability. For example, Guilherme Suarez-Kurtz, a prominent investigator in the field of pharmacogenetics, coordinator of the National Network of Pharmacogenetics and Pharmacogenomics, senior member of the Brazilian Academy of Sciences, and researcher at the Instituto Nacional do Cancer (INCA or National Cancer Institute) in Rio de Janeiro, commented recently on the application of algorithms to estimate warfarin dosage to research on Brazilian populations.

… warfarin dosing algorithms that have a ‘race’ term defined by criteria prevalent in a given region or country (e.g., the United States OMB) are unlikely to be applicable worldwide, especially in extensively admixed populations, such as Brazilians. On a broader perspective, one may ask whether the global population diversity may be captured by inserting ‘race’ terms in PGx algorithms that are sufficiently ‘friendly’ to be adopted by the practicing prescriber?

(Suarez-Kurtz, 2010, pp. 3–4).

Authors such as Suarez-Kurtz and the geneticist Sergio Pena have emphasized the mixed race question in Latin America, and especially in Brazil, at the same time criticizing the racial perspective in pharmacogenomics (referred to by the euphemism ‘population-based drug development and prescription’) (see Suarez-Kurtz and Pena 2006a, b; Suarez-Kurtz, 2010; Pena, 2011; Pena *et al*, 2011). They link the production of knowledge in pharmacogenomics to such a broad question as inequalities in public health policy, although these moral claims, somewhat differently to the United States, rest on the need to demolecularize race and genomics as the rest of the article examines. Looking at Latin America, especially with regard to race mixing, these scientists argue that the region may provide a counterpoint (because of its high level of ‘genetic admixture’) to thinking of pharmacogenomics in terms of the world as a whole.

The aim of this article is to discuss issues that have been the subject of recent debates in the field of pharmacogenomics, especially those concerning the use of race/color categories, with resulting effects on the genomic profile of human populations. On the basis of a Brazilian case, we will examine how pharmaceutical technologies, generally developed in North America or Europe, and their prescribed uses (as in the case of ethno-racial categories) circulate globally. In the first place, we situate pharmacogenomic research in Brazil in the context of continuities in the historical studies on the genomic diversity of Brazilian populations, which notably increased in the 1990s and the early 2000s. Then we argue that a central point of pharmacogenomic research in Brazil has been to emphasize the specific historical trajectory of the country that led to extensive racial mixing and the resulting impact on issues as diverse as the use of algorithms for the administration of drugs, the use of color/race or ethnic categories in genomic research, and the directions of public policy in the area of health. The second theme, deployed as a case study, will be the exploration of research on the anticoagulant warfarin by Brazilian pharmacologists and geneticist. We then situate the criticism of the focus on race that has emerged from pharmacogenomic research in Brazil in relation to the broader debates in the anthropology of science literature on genomics research on pharmacogenomics as well as studies of admixture. We see parallels and differences in the way that these debates have been critiqued and theorized with the Brazil context, where there is both what might be seen as incorporation and simultaneous rejection of transnational genomic research that makes use in different ways of race and color categories.

## Biological Diversity of the Brazilian Population

In order to understand recent discussions about race/color categories in the context of research in pharmacogenomics in Brazil, it is essential to be aware of their background, that is, the scientific work on the biological diversity of the Brazilian population that has been done over the last decade. A number of the works by Suarez-Kurtz on pharmacogenomics refer to this literature, especially to the research by the geneticist Sergio Pena since 2000. As we will see in this section, genomic interpretations of the biological make-up of Brazilians relate to perspectives that go back half a century to the origin of human population genetics in Brazil.

In Brazil, human population genetics became established as a specific field of investigation in the 1950s, when research groups were formed in several Brazilian institutions. The support of the Rockefeller Foundation was crucial, since, among other initiatives, it funded a number of trips to Brazil in the 1940s and 1950s by the geneticist Theodosius Dobzhansky of Columbia University (Glick, 1994; Santos and Maio, 2004; Souza *et al*, 2013; Souza and Santos, 2014). In partnership with the Biology Department of São Paulo University (Universidade de São Paulo – USP), and especially with the Brazilian scientist André Dreyfus, Dobzhansky carried out field studies in various parts of the country, gave courses and took part in training young Brazilian geneticists. Many of them would go on to write their doctoral dissertations on *Drosophila* genetics. In the 1950s, some of these geneticists turned to the emerging field of human population genetics. Also with the support of the Rockefeller Foundation, investigators who would become important figures in Brazilian genetics, such as Francisco Salzano, Newton Freire-Maia and Pedro Saldanha, went to the United States for postdoctoral training, especially at the Department of Human Genetics of the University of Michigan, with James Neel. Moreover, in the early 1960s a group of Brazilian geneticists earned their doctorates at the University of Hawaii with the population geneticist Newton Morton (Souza and Santos, 2014).

This first generation of researchers in human genetics formed in the 1950s and 1960s mainly followed two lines of research (Souza *et al*, 2013; Souza and Santos, 2014). One was the genetics of indigenous populations, in which the work of Francisco Salzano, in partnership with James Neel, stands out (see Santos, 2002; Lindee, 2004; Souza and Santos, 2014; Santos *et al*, 2014b). Their interest centered on genetic evidence about the peopling of the Americas, and study of the processes that produce human biological diversity (so-called micro evolutionary processes). A second line that was prominent at the beginning of human population genetics in Brazil, and is of particular interest for this study, concentrated on understanding, from the viewpoint of genetics, how the Brazilian population was formed from the interaction of the so-called main ‘racial stocks’ (that is, European, African and Amerindian) (see Salzano and Freire-Maia, 1970).

On this second line, through analysis of the frequency and distribution of alleles of the classic genetic markers (the ABO blood groups, Rh, Diego factor and serum proteins, as well as gamma globulins), interest largely focused on the investigation of “racial admixture” (Souza *et al*, 2013; Souza and Santos, 2014; Santos *et al*, 2014a). Influenced by the neo-Darwinian perspective that increasingly dominated human population studies, and, at the same time, infused with racialized views, these studies were largely aimed at analyzing processes of genetic flow between the ‘parental populations’. The principal geneticists who developed studies of ‘racial admixture’ in the 1950s and 1960s, such as Pedro Saldanha, Newton Freire-Maia, Ademar Freire-Maia, Eliane Azevedo, Henrique Krieger and Francisco Salzano, believed that Brazil presented particularly propitious conditions for genetic studies, in view of the genetic diversity, the social, demographic and epidemiological conditions of the country. Like other regions where there was intense immigration and racial mixing, such as Hawaii (Anderson, 2012), Brazil was seen as a great “racial laboratory”, as the Brazilian geneticist Newton Freire-Maia entitled one of his books (*Brasil*: *Laboratório Racial*; *Brazil*: *Racial Laboratory*), published in 1973. In the opinion of Salzano and Freire-Maia, as they wrote in a book published in 1970, Brazilian populations “present the geneticist and anthropologist an excellent opportunity for the study of complex and fascinating problems”, in view of “the extreme variety of their original ethnic groups, the widespread miscegenation, and their distribution in all kinds of environmental conditions” (Salzano and Freire-Maia, 1970, p. xv).

Genetic research on human populations went through a fundamental methodological transition during the 1980s, when the classic genetic markers were increasingly replaced by DNA markers. What happened in Brazil was that the two agendas described above, which already dominated research in the genetics of human populations (that is, genetics of indigenous populations and studies of the biological diversity of the Brazilian population) were methodologically updated in the form of genomics. The same themes continued to be central to the research agenda of human genetics in Brazil.

In the early 2000s, the publication of two articles that used a genomic approach may be considered a watershed in the investigation of human biological diversity in Brazil (Alves-Silva *et al*, 2000; Carvalho-Silva *et al*, 2001). These publications, which were based on analysis of mitochondrial DNA markers and the Y chromosome, were by a group coordinated by the geneticist Sérgio Danilo Pena, of the Federal University of Minas Gerais (*Universidade Federal de Minas Gerais*). The researchers obtained from paternity testing clinics genetic samples of approximately 250 men self-classified as ‘White’ who came from different regions of the country. Analyzing polymorphisms of the Y chromosome in these samples showed that a vast majority of markers could be identified as European in origin, with a very low frequency of markers originating in sub-Saharan Africa, and no Amerindian genetic contribution. On the other hand, the analyses of mitochondrial DNA gave a much more complex picture, with 33 per cent Amerindian genetic contribution and 28 per cent African contribution, a surprisingly high African and Amerindian percentage in the matrilines of the White men studied. As the authors noted in a popular science article, this pattern of differential reproduction (with patrilineages verified through the Y chromosome predominantly of European origin, and matrilineages verified through mitochondrial DNA as largely African and Amerindian) made much sense in view of the history of the colonization of Brazilian territory from the sixteenth century: “the first Portuguese immigrants did not bring their wives, and historical records indicate that they rapidly began a process of miscegenation with indigenous women. With the arrival of slaves, beginning in the second half of the sixteenth century, the miscegenation was extended to African women” (Pena *et al*, 2000, p. 25). In the geneticists’ perspective, this research demonstrates how genetically mixed is the sample of (self-classified) White men, since the majority of the matrilineages studied (approximately 60 per cent) had an Amerindian or African origin.

As well as research using mtDNA and the Y chromosome, Pena’s group, early in the 2000s, carried out investigations based on autosomal markers, that is, genetic traits located in the cell nucleus (Parra *et al*, 2003). In a study entitled “Color and genomic ancestry in Brazilians” a group of approximately 170 people from Queixadinha, a community in the interior of Minas Gerais, in the southeast region of the country, was investigated. Possibly the principal finding of this study was that there was no correspondence between the morphological and the biological classification of this sample. The researchers noted that a large overlap made it difficult to distinguish the genomic characteristics of persons morphologically classified as ‘White’, ‘Brown’ or ‘Black’. In contrast, a comparison of the genetic characteristics of three other groups (Africans of São Tomé, Amazonian indigenous populations and Portuguese) showed correspondence between their morphological and genetic characteristics. The authors concluded: “Our data suggest that in Brazil, at an individual level, color, as determined by physical evaluation, is a poor predictor of genomic African ancestry, estimated by molecular markers” (Parra *et al*, 2003, p. 177). A study along similar lines carried out by the Pena group a few years later with samples from the city of São Paulo reached a similar conclusion: “These results corroborate and validate our previous conclusions using ancestry-informative markers that in Brazil at the individual level there is significant dissociation of color and genomic ancestry” (Pimenta *et al*, 2006, p. 190).

Following the example of the group from the Federal University of Minas Gerais, coordinated by Pena, since the early 2000s there has been intense production of studies on the genomic diversity of the Brazilian population by research groups from various other Brazilian institutions. For example, there are groups affiliated with the Federal University of Rio Grande do Sul, the University of São Paulo, the Federal University of Paraná, the Catholic University of Brasília and the Federal University of Pará, among others (see Santos *et al*, 2014a). The investigations by these research groups located in different parts of the country, from north to south, and based on samples collected in their respective regions, have resulted in intense interest in understanding not only the general aspects (on a national scale) but also how local/regional dynamics have been involved in the make-up of Brazilian populations.

We can cite as an example, the studies of the research group from the Federal University of Rio Grande do Sul on the formation of the so-called ‘Gaúcho’ population (that is, from the state of Rio Grande do Sul) (see Kent and Santos, 2014). The interpretive models of Pena and his collaborators tend to focus on the nation as a whole, so at times analyses based on populations that are more socially and geographically bounded have resulted in discrepant interpretations. In the case of Rio Grande do Sul, Guerreiro-Junior *et al* (2009, p. 1) indicated that their findings “emphasize the need to consider in Brazil, despite some general trends, a notable heterogeneity in the pattern of admixture dynamics within and between populations/groups”. Zembrzuski *et al* (2006), by their turn, argue that in Rio Grande do Sul, a state that has been the scene of intense European colonization, and where there is less miscegenation than in other parts of the country, there is, indeed, a close association between genomic profile and physical appearance.

In spite of different interpretations of certain aspects of the biological diversity of the Brazilian population, scientific studies done by the various research groups tend to agree that in Brazil there is great genetic heterogeneity at both the national and regional levels.^2^ Moreover, this diversity has been interpreted by the geneticists as derived from the interaction among populations of European, African and Amerindian origin that, depending on the region of the country, each with its own particular history, resulted in genomic profiles with particular characteristics. Therefore we can affirm that in the studies on biological diversity in this genomic era there is marked continuity with perspectives that predominated at the origins of population genetics in the country in the 1950s and 1960s. We see in both eras an effort to emphasize genetic diversity of the population in terms of national difference and the uniqueness of the South American region. At the same time there is also marked continuity in the effort to highlight the transnational utility of Brazil as a ‘racial laboratory’ for genetic research (Salzano and Freire-Maia, 1970; Freire-Maia, 1973; Santos *et al*, 2014a, b; Souza and Santos, 2014), while also continuing to make use of what are assumed to stable parental population categories referenced through the notion of tri-hybrid ancestries.

Starting approximately in 2005, studies on the genetic profile of the Brazilian population became increasingly made part of investigations in the field of pharmacogenomics. This is evident in the work of Suarez-Kurtz and Pena, published in 2006, entitled, “Pharmacogenomics in the Americas: Impact of genetic admixture”. In this work they affirm:

Because interethnic admixture is either common or increasing at a fast pace in many, if not most populations, extrapolation on a global scale of pharmacogenomic data from well-defined ethnic groups is plagued with uncertainty. Intra-ethnic diversity adds complexity to the scientific appraisal, regulatory decisions and, eventually, prescribing of drugs purportedly targeted to a given ‘race’ or ethnicity. Pharmacogenetics/genomics has the potential to benefit people worldwide and to reduce the health disparities between developing and developed nations. This goal is unlikely to be achieved by relinquishing the notion of personalized drug therapy tailored to individual genetic characteristics – the original promise of pharmacogenetics – in favor of a model (pharmacogenomic?) of population-based drug development and prescription, with all its potential pitfalls, especially when extended to admixed populations in developing or developed nations.

(Suarez-Kurtz and Pena, 2006a)

In recent years, Suarez-Kurz and Pena have carried out joint studies (Suarez-Kurtz *et al*, 2007, 2010a, b; Pena *et al*, 2011; Sortica *et al*, 2012), some of which we will comment on later in this work. What we should like to stress here is the focus on a model of genetic diversity that emphasizes the topic of admixture and how this is something to be considered not only in the conduct of research, but also in plans to apply knowledge derived from pharmacogenomics to public policy. Quoting Pena (2007), Suarez-Kurtz (2010) speaks of the existence of three models for the evolution and structure of genetic diversity in the human species. The first, referred to as typological, that prevailed in the nineteenth and the beginning of the twentieth century, divided the human species into ‘races’ conceived as different from one another and internally homogeneous; the second, called ‘population’, that prevailed from the middle of the twentieth century, is a product of the Neo-Darwinian syntheses of the first decades of the century. According to Suarez-Kurtz (2010), “both models are poor descriptors of admixed populations, since genetic admixture is best modeled as a continuous variable” (p. 1).

In this sense, the model the pharmacologist considers the most adequate to describe the biological variability of the human species, and therefore that most appropriate for use in pharmacogenomic studies, is the ‘individual singularity’ model. Quoting Patrinos (2004), Suarez-Kurtz (2010, p. 1) argued that “each person must be treated as an individual … rather than as an exemplar of a race”. Yet while this is a model of individuality that, in Brazil and elsewhere, is informed by the promissory dimensions of pharmacogenetic research as ‘personal medicine’, it also stands in contrast to Fulwilley’s critique of personalized pharmacogenomics in the United States in which admixture is read through histories of hypodescent and biracial hybrids (Fullwiley, 2007). In Brazil such a model, in the context of pharmacogenomics research, is instead situated and emerges from complex clinal understandings of inherent individual genetic ancestral variability and mixture that is linked in Brazil to quite to different histories and politics of race, color and national identity (see for instance, Maio and Santos, 2010; Gibbon *et al*, 2011; Souza and Santos, 2014; Santos *et al*, 2014a, b).

As we shall see in the next section, the pharmacogenomic research that Suarez-Kurtz is carrying out on warfarin has been strongly influenced by this perspective of ‘individual singularity’ and thus is distanced from algorithms that define dosage based on the notion of ‘race’.

## Warfarin and Miscegenation

Suarez-Kurtz, who has been affiliated since 1960 with the Federal University of Rio de Janeiro (*Universidade Federal do Rio de Janeiro*, or UFRJ), has dedicated his working life to the fields of physiology and pharmacology. From the start of his career he traveled extensively abroad for study and research. He would continue the research that he developed at institutions in France, England and the United States when he returned to Brazil, sometimes adapting it to local conditions. In the 1970s and 1980s, he continued in Brazil the studies of muscular physiology, using crayfish as the experimental model, that he had begun abroad, substituting a small blue crab that scuttles along Brazilian beaches. Studies on frogs, funded by the American Association for Muscular Dystrophy, were continued in the UFRJ laboratories with mice and opossums. In his search to unite creativity (expressed by designing novel experiments) with continuity (by re-adapting the experiments begun abroad to local conditions), he developed an approach that incorporated autochthonous objects as essential elements in universal models. In one of the interviews he gave us,^3^ Suarez-Kurtz defined his search for a “strategic advantage” as, for him, an alternative to what might be considered by Brazilian scientists mere repetition of what was already done abroad.

In the early 2000s, when Suarez-Kurtz began research in pharmacogenetics at the National Cancer Institute (INCA) in Rio de Janeiro, he used this creative/adaptive approach in new circumstances. He made the Brazilian population the object of his research in pharmacogenomics, but Suarez-Kurtz chose not to accept its distinctive character as a variation on a general paradigm, but rather to question the viability of a model accepted by pharmacogenetics as universal. It was the perception of the uniqueness of the autochthonous object as something innovative and indispensable for the development of pharmacogenetics that led him to affirm that this was no longer a ‘strategic’ choice for discussion, but a specific with global relevance. This approach that characterizes the research on pharmacogenomics of warfarin carried out at INCA reflects strategies that have long been embedded in genetic research in Brazil, as we have already discussed, with their focus on both aligning with transnational research agendas and establishing Brazil as uniquely different and important context for study (Maio and Santos, 2010; Gibbon *et al*, 2011; Souza and Santos, 2014; Santos *et al*, 2014a, b).

Warfarin, which has been in use since the 1950s, combines, according to Suarez-Kurtz, various characteristics that make it an interesting model for study: (i) it is the most commonly prescribed oral anticoagulant in Brazil and worldwide, (ii) there is large inter-individual variation in the required dosage, (iii) its therapeutic index is narrow and incorrect dosage, especially during the initial phase of treatment, carries a high risk of bleeding or failure to prevent thromboembolism, and (iv) a reliable biomarker, the international normalized ratio, is available for quantifying warfarin’s anticoagulant effect (Perini *et al*, 2008, p. 726; Suarez-Kurtz, 2010, p. 6). Moreover, from the viewpoint of genetics, its inheritance is relatively simple, as polymorphisms in two genes (*CYP2C9* and (*VKORCI*) have been identified as associated with the clinical response to warfarin (Suarez-Kurtz, 2010, p. 6).

In the article entitled “Pharmacogenetics of warfarin: development of a dosing algorithm for Brazilian patients”, Perini *et al* (2008) investigated the predictive capacity of an algorithm for dosing warfarin, comparing a total of 390 patients self-classified as ‘White’, ‘Intermediate’ or ‘Black’, from a cardiology hospital in Rio de Janeiro^4^. The analyses indicated a wide variation in warfarin dosage according to color/race groups (from 5 to 75 mg/week), with differences between ‘White’ (lower dose) on one hand, and ‘Black’ and ‘Intermediate’ on the other (Perini *et al*, 2008, p. 722). Results showed that patients of the three color/race groups varied in allelic frequencies and in the distribution of genotypes of the two polymorphisms, and that the genotypes were associated with the dose of warfarin, although with wide overlap in the required dose (p. 724). The authors also estimated what would be the most appropriate algorithm to predict the warfarin dose for this group of patients. While in the univariate analysis they observed that the color/race variable was statistically significant, in the multivariate regression analysis this variable was not retained. As to the relationship between the predicted dose and the required dose, Perini *et al* (2008, p. 725) argued that there was overlap among the color/race groups. The authors also emphasized that, from a statistical point of view, the algorithm that does not include the color/race variable shows better results when compared with others developed in other parts of the world. In comparison with an algorithm developed in the United States they commented:

… self-reported race/color was not a significant predictor of warfarin requirements in our patient cohort, despite the significant differences in the maintenance warfarin dose across the race/color groups. This contrasts with a recently published warfarin dosing algorithm derived from a large cohort of US patients that included ‘African-American race’ as a significant covariate. This discordance may be related to a number of factors including (i) our observation that *VKORC1* and *CYP2C9* genotype differences in warfarin dose requirements exist independent of self-reported race/color among Brazilians …, (ii) the greater proportion of European genetic ancestry among black Brazilians compared with African Americans, and (iii) the poor correlation of self-reported race/color with genetic ancestry among Brazilians

(Perini *et al*, 2008, p. 726).

In Brazil, Perini *et al* (2008) concluded, the most appropriate option would be to consider the individual portion of African genomic ancestry, independent of color/race self-classification. According to them, “… because of the heterogeneity and extensive admixture of the present-day Brazilian population, extrapolation of data derived from well-defined ethnic groups is clearly not applicable to the majority of Brazilians” (p. 722).

The work of Perini *et al* (2008) did not include information on the genetic profile of the individuals analyzed. The next step in the research coordinated by Suarez-Kurtz was to take into consideration genomic profiles, using for this purpose samples collected through the National Network of Pharmacogenetics and Pharmacogenomics (REFARGEN), created in 2003 by the initiative of Suarez-Kurtz himself. This database includes 1037 Brazilians considered healthy recruited in four regions of the country. As well as being self-classified by color/race (as ‘White’, ‘Brown’ or ‘Black’) the genomic ancestry of these individuals, reported in Suarez-Kurtz *et al* (2007), was evaluated using a panel of 40 genomic markers (in this case INDELS, or Insertion-Deletion polymorphisms) developed by Sergio Pena’s team (Bastos-Rodrigues *et al*, 2006). It is important to note that this panel of genomic markers is based on 40 INDELS validated in samples from the HGPD–CEPH Human Genome Diversity Cell Line Panel (see Bastos-Rodrigues *et al*, 2006). In that publication the authors write that the methodology “confirmed the partition of worldwide diversity into five genetic clusters that correspond to major geographical regions” (p. 658), that is, Africa, Europe, America, Oceania, Middle East, East Asia and Central/ South Asia. In this sense, the use of this panel of markers by the Brazilian pharmacogenomic research might be seen to non-geneticists as contributing to the assumption that there are stable continental ancestry groups.^5^

According to Suarez-Kurtz (2010, pp. 2–3), the principal genomic aspects identified in the REFARGEN samples were the following: (i) most individuals have significant degrees of European and African ancestry, whereas a sizable number display also Amerindian ancestry; (ii) the average proportions of European ancestry decreased progressively from ‘White’ (0.86), to ‘Brown’ (0.68) and then to ‘Black’ individuals (0.43), whereas the opposite trend was observed with respect to African ancestry; (iii) Amerindian ancestry was relatively constant across the three groups, ranging from 0.07 and 0.09. On the basis of this characterization, it was argued that, in the Brazilian case, European and African ancestries have greater impact on the frequency of polymorphisms of pharmacogenomic relevance, as compared with Amerindian ancestry. While making use then of genomic markers that could be argued to reproduce stable categories of ancestry, population or even race, we find Brazilian pharmacogenomics incorporating the category of ‘Brown’ into their genomic findings and most importantly explicitly emphasizing the clinal nature of genetic ancestry. That is as Suarez-Kurtz stated that “the individual proportions of European and African ancestry vary over wide ranges as a *continuum* across the Color categories of the Brazilian Census” (Suarez-Kurtz, 2010, p. 3).

Using the REFARGEN samples, Suarez-Kurtz *et al* (2010a, p. 1258) carried out an investigation with the objective of evaluating the effect of three variables (geographic origin, self-reported color/race and genetic ancestry) on the distribution of four *VKORC1* single nucleotide polymorphisms and their haplotypes in the Brazilian population. They also attempted to compare the results from the Brazilian samples with collections from Europe and Africa (specifically Portugal, Angola and Mozambique), with the intent to “explore the genetic variability of a major pharmacogenomic target in the population of Brazil, having as reference two of its most important ancestral roots” (ibid.). The main conclusion was the following:

The evidence presented here extends to a major pharmacogenomic target, *VKORC1*, the notion that the diversity and heterogeneity of the Brazilian population is not captured by self-identified race/Color classes. The pattern of distribution of *VKORC1* polymorphisms among Brazilians is best described as a continuum, reflecting the individual proportions of European and African ancestry

(ibid., p. 1265).

In the authors’ opinion, care should be taken when extrapolating pharmacogenomic information derived from ‘other ‘racial/ethnic/continental’ groups’ to the Brazilian context. They question the clinical use in Brazil of algorithms for warfarin dosage that do not take into consideration the biological diversity of the Brazilian population.^6^

Going beyond strictly scientific articles, both Suarez-Kurtz and Sergio Pena have published opinion pieces directed at the general public. In these articles, they stressed how, in their interpretation, pharmacogenomics and the genetic diversity of the Brazilian population relate to public health policy. In 2009, Suarez-Kurtz published an editorial in *Cadernos de Saúde Pública/Reports in Public Health*, one of the leading journals in public health in Latin America that is affiliated with an institution (Oswaldo Cruz Foundation, or *Fundação Oswaldo Cruz*) of the Ministry of Health. This journal is one of the main Brazilian periodicals that carry academic papers referring to the National Public Health System (SUS or *Sistema Único de Saúde*). In Brazil, this system includes all areas of health, from outpatient care to organ transplants, with the intent of providing complete, universal and free care for the whole population of the country. In this editorial Suarez-Kurtz (2009) wrote:

The results [of our research] showed that the heterogeneity of our population must be dealt with as a continuous variable, which cannot be adequately represented by arbitrary ‘race/color’ categories. In a PGx-informed context, this implies that each person must be treated as an individual rather than as an ‘exemplar of a race’, and that the notion of ‘race-targeted’ drugs is unacceptable, especially in the case of admixed populations

(p. 1650).

The results of the research in pharmacogenomics by the INCA might be seen as reflecting the medication policy of the National Health System, which is carried out by the “National Policy for Pharmaceutical Assistance”. This policy was declared by the National Health Council in 2004, and it is an integral part of the “National Health Policy”. One of the purposes of the “National Policy for Pharmaceutical Assistance” is the “implementation throughout government sectors, and especially through the Ministry for Science and Technology, of public policy for scientific and technological development, involving research centers and universities, with the objective of promoting technological innovation [in the area of medication] that serves the national interest and the necessities and priorities of SUS” (CNS (Conselho Nacional de Saúde), 2004). Therefore, there are close links between pharmacogenomic research and the wider area of public health policy in Brazil. There are though key difference here compared with the United States where moral claims around public health and genomic research has focused on the rights and needs of ‘underserved’ racialized groups and questions of social justice (Lee, 2005) as we discuss below. In Brazil, the current SUS priority of health care access for all means that the inequities at stake in Brazil are framed (for the time being at least) around universalizing access to drugs and services rather than the underserved needs of specifically defined racialized groups or populations.^7^

## Placing in Broader Context

Fullwiley (2007) has analyzed how public policy in the United States has molded health research in such a way that, in order for the research to be funded, racial categories have to be at the base of methodological designs. It is obligatory, when researchers construct their samples, to specify a racial group to which a donor belongs, for example: *African American*, *Native American*, *Mexican American*, *Asian*, *Caucasian* or *Hispanic*, even though, as Fullwiley observes, these classifications are necessarily ambiguous. The author writes that, in 1990, two government institutions, the US Department of Energy and the National Institutes of Health (NIH) were major sponsors of the international Human Genome Project. At that time, the great expectation was that the Project would lead to the development of genetic therapies. However, according to Fullwiley, current interest in our so-called ‘Genomic Era’ has, in the United States, turned away from genetic therapy and toward generating knowledge about specific pharmacogenetic characteristics, based on the genetic profiles of samples whose ethno-racial classification is a central parameter.

Fullwiley (2007) does not attribute this new perspective to a deliberate ‘racist posture’ on the part of American government institutions, such as NIH, involved in health research. According to her analysis, the change is the result of the growing influence of social movements that have organized politically to gain recognition (see Epstein, 2007). The author does not question the legitimacy of the scientists’ interest in human variation, but she deplores the ambiguous way in which racial categories have entered the formulation and analysis of research in pharmacogenetics. The way in which racial taxa are filled in at the time the samples are set up is extremely fluid. The researchers may use classifications that they create themselves, in which case the scientists, observing the appearance (phenotype) of the patient, place him or her in one of the assigned racial groups, or they may rely on the self-attribution of the patient, or choose an attribution based on ancestry, or, finally, they may mix various criteria in setting up a single sample.

Fullwiley shows how, in pharmacogenomic research, differences in appearance that previously were referred to as race are replaced by certain genes, in a process that she calls the “molecularization of race”. Through this process, race is assigned by means of “a substance discernible at the molecular level” (Fullwiley, 2007, p. 4). She observes that the scientists she interviewed at two major genomic research laboratories in California were preoccupied with obtaining ‘racially pure’ DNA samples, meaning that for certain research projects samples from donors with a history of ‘racial mixture’ in the past three generations are excluded. The author also shows that ‘White’ and ‘Black’ are considered ‘opposites’, so that it is expected that a high frequency of a gene among White individuals will correspond to a low frequency of the same gene among Black individuals. When this expectation is not fulfilled, the explanation is that the persons recruited as donors were not sufficiently ‘pure’ in racial terms.

Another work that is relevant to understanding how the idea of race made its way to the center of pharmacogenomic studies is one that Bliss (2011, 2012) has written on what she calls the “genomic elite”, that is, leading researchers at major research institutions in the field. The author gives the term “reflexive biosociality” to the fact that these researchers, while at the same time considering race a “social construct”, base their scientific activity on racial criteria – not to give support to a “racist racial biomedicine” (ibid., p. 1024) – but to construct an antidote to racial discrimination. According to her, “these researchers feel that subject self-identification is enacted in the interest of antiracist values filtered through the constraints of genomic protocols” (ibid.). Bliss’s approach differs from that of Fullwiley in terms of the extent to which the use of ‘racial taxonomy’ seems to be problematic for the scientists she interviewed.

Therefore, while Fullwiley encounters scientists who, while committed to addressing health disparities and injustices in their work, seem to be somewhat less critical about the use of racial terminology to qualify their samples, Bliss (2011, 2012) claims that her interviewees are engaged, and that is the explicit reason why they adopt ‘race’ as a scientific parameter. Like some of the researchers Fullwiley encounters, they are scientists who, at some moment in their lives were sensitized against racism, either because they descended from specific ethnic groups, or recognize that they belong to one of them, or because of some marked event in their life history. Thus their views, which oppose the idea of ‘race’ as a substance or type with an insoluble group of defining characteristics, may be compatible with the intention, through scientific research, of giving visibility to discriminated segments of society, or of creating conditions for their inclusion. The investigators feel that genomics can take a central role in this, and “make exceptions for use of racial taxonomy as a heuristic in research that covers health disparities” (Bliss, 2011, p. 1024). Like Fullwiley, Bliss points out the importance of the idea of ‘racial purity’ in research, as well as the ambiguity in the construction of samples that mix alternative criteria, that is genealogy, self-attribution and classification by the researchers.

Michael Montoya’s work has been more explicitly concerned with question of admixture in Latin America in the context of genomic research of Type 2 Diabetes among Mexican–Americans (2007, 2011). He points out how racial purity becomes displaced by concerns about the need for ‘homogeneity’ in Mexican–American population samples, even as this also entails a certain naturalization of the social order that reflects somewhat entrenched patterns of inequality and subjugation. Nevertheless, like Bliss, he encounters scientists who are reflexively and politically aware of the importance of the ethnoracial taxonomies in the context of their research. He notes in fact the ways that ethnic identities [and histories] are pressed “into the service of the biological” in research and development of the genetic science and medicine in this context. This is a process he terms “bioethnic conscription” that frequently involves, he points out, a slippage between a simple pragmatic description of ethnic-racial labels to identify groups to one of the attribution of qualities to those groups (Montoya, 2007, p. 95). For Montoya, such practices reflect and inform the “infrastructure of racialization” (Lee, 2005, 2012) in the United States that is increasingly being incorporated into transnational research.

How does our case study on Brazil fit in these theoretical and ethnographic contexts we outlined in this article? On the one hand, we suggest that, far from witnessing a ‘molecularization of race’ or admixture as Fuwilley and Montoya outline, the Brazilian research leads to questioning the validity, for Brazilian (‘racial’) reality, of the parameters constructing this ‘infrastructure’. This is because the Brazilian research in pharmacogenomics analyzed in this article tends to work toward the ‘demolecularization of race’, which is justified by the narrative that places miscegenation as the distinctive and central element in the biological formation of the Brazilian population. Thus, if ‘races’, as discrete units, are seen as an “organizing principle … as a natural choice and referent for categorizing humans for many working in this field [of pharmacogenomics]” (Fullwiley, 2007, p. 4) in the United States, in the Brazilian case what is foregrounded is the non-relevance of and an implicit critique of race as heuristic device in pharmacogenomics research.^8^

Yet, at the same time, it must be acknowledged that the effort of ‘demolecularizing race’ by pharmacogenomics in Brazil passes through a process in which, using Lee’s (2005) term (p. 2134), “bodies and bodily materials are nonetheless racialized institutionally”, even though this relates in Brazil less to the development of niche markets for new pharmacogenomic targets than the context in which transnational research, collaboration and publication take place. We can perceive in the work of Suarez-Kurtz and his collaborators the use of a heuristic strategy where it is clear that, even while there is a critique of the use of racial categories in genetic research, there is necessary use value and incorporation of categories and taxonomies that are somewhat race like; we call this ‘racialize to deracialize’. This is particularly evident in the investigation on the algorithm for warfarin dosage. In the case of this study, the investigators started with the presupposition that a ‘racialized’ classification of the subjects, with the categories ‘White’, ‘Intermediate’, and ‘Black’, would be used as an explanatory variable in the algorithms defining dosage, but later concluded that this categorization was not a relevant variable. If, in general, investigations to define algorithms aim to precisely identify and quantify the participation of the so-called ‘independent variables’ (like age, sex, weight, clinical characteristics and so on) in the expression of the outcome (in this case the dose of warfarin), the strategy followed in the work of Perini *et al* (2008) is to point out the unimportance of ‘race’ membership as an explanatory variable. Thus we have the ‘molecularization of race’, followed by its ‘demolecularization’.^9^

There are some important parallels in this conclusion with the work of Fujimura and Rajagopalan examining the use of a particular software for calculating continental ancestry in the context of Genome Wide Association Studies in the United States. They suggest that such techniques, that simultaneously produce “different kind of populations” and “population differences”, entail what they describe as “genome geography”. Outlining this further they state:

The concept of genetic ancestry and the practice of genome geography rely on old discourses, but they also incorporate new technologies, infrastructures, and political and scientific commitments. Some of these new technologies provide opportunities to change some of our institutional and cultural forms and frames around notions of difference and similarity. Nevertheless, we also highlight the slipperiness of genome geography and the tenacity of race and race concepts

(Fujimura and Rajagopalan, 2011, p. 5).

Similarly even though the Brazilian geneticists situate themselves in terms of a radical rejection of the use of categories of color and race in the calculation of dosing algorithm for wafarin, it is important to consider how the work of ‘racializing to de-racialize’ is accomplished using techniques to characterize human genetic diversity that involve what might appear to be, stable categories of continental ancestries or race.^10^

The tension that we suggest is central to the practice of racializing to de-racialize around incorporation, and rejection of categories of race or continental ancestry in pharmacogenomic research is nowhere more apparent than in the context of transnational research that the Brazilian geneticists are engaged with and what might be described as the political economy surrounding the publication of research.

Considering the circuitous route traveled by scientific data after its production in the pharmacogenomic laboratories in Brazil, Suarez-Kurtz commented more than once in the interviews he gave us about his participation in international research consortiums (that is, multi-centric studies) on the pharmacogenomics of warfarin. These studies involve comparative analyses of hundreds or thousands of samples from various countries. An example is the case of the paper, “Estimation of the warfarin dose with clinical and pharmacogenetic data”, published in the *New England Journal of Medicine* by the IWPC (International Warfarin Pharmacogenetics Consortium) *et al* (2009) and composed by more than a hundred authors.^11^ The study involved the use of clinical and genetic data from 4043 patients, with the intent of developing and testing an algorithm for estimating the appropriate warfarin dose. Suarez-Kurtz said, in our interview, that only a small portions of the Brazilian samples sent were utilized in the multi-centric study, and most of the data from individuals classified as ‘Intermediate’ were not included. In fact, in the article the data that are analyzed are those referred to as ‘White’, ‘Asian’ or ‘Black’, whereas ‘Mixed or missing data’ constitute a combined category not used in the analyses (ibid.).

That is, when they enter the ‘political economy’ of publications in pharmacogenomics that involve extensive international collaboration, often the Brazilian samples, especially those of ‘mestizos’, are not included because they seem not to fit the required ‘racial framework’. The scientists that Montoya worked with on the genetics of Type 2 Diabetes also confronted a parallel yet different political economy of publications in their efforts to make their data publishable and of universal relevance without being ghettoized as being only relevant to Mexican–Americans (2007, 2011). Along these lines, if in the perspective of Brazilian pharmacogeneticists the data produced in the country may serve to produce a critique of the universalization of data derived from specific contexts (as is the case of warfarin algorithms developed in the United States), what the example of the multi-centric research seems to indicate is that many Brazilian samples are not sufficiently ‘racially defined’ to enter the body of production of a universal and transnational science.

## Final Considerations

In a talk he gave at a seminar on pharmacogenomics held in Puerto Rico in 2010 (with the title “Pharmacogenomic implications of genetic admixture in Brazilians”), one of the many given on his research at international forums in recent years, Suarez-Kurtz reiterated the conclusion that he has often repeated in his publications: “The heterogeneity of the Brazilian population must be taken into account in the design and interpretation of PGx (pharmacogenomic) clinical trials …” This time Suarez-Kurtz illustrated his remarks with three groups of images. The first, (surprisingly) along with a quotation on Brazil by the French count Arthur Gobineau (a leading opponents of miscegenation in the nineteenth century), showed the painting “The Workmen” (1933) (*Os Operários*), by Tarsila do Amaral. This work, by one of the most famous Brazilian modernist painters, depicts the themes of miscegenation (from a positive perspective), industrialization and social transformation in Brazil (and especially in São Paulo) in the early decades of the twentieth century.^12^ The second group of images was, in a way, a re-reading of Amaral’s work. It presented 14 faces, with the most varied skin color, of personalities who are famous nowadays in Brazil in the fields of music, sport, literature, architecture, cinema and medicine. The third group was composed of two photographs of football players (one from 1970 and one from 2006) that may be interpreted as referring to Suarez-Kurtz’s approach to pharmacogenomics by contrasting models based on discrete racial categories with those that emphasize the notion of a continuum. In the 1970 photograph, we see two players with distinct skin color, one Black and one White: one Brazilian (Pelé) and one English (Bobby Moore), shaking hands at the end of the 1970 World Cup. In the 2006 photograph, five players form a spectrum from the whitest on the left to the blackest on the right. Bringing together these groups of images is a quotation from *Del Amor y Otros Demonios* (Of Love and Other Demons), a book by the Colombian Nobel Prize Winner Gabriel Garcia Marquez: “At my age and with so much mixed blood, I no longer know for sure where I belong. Nobody knows it in these lands … and I believe that it will take centuries to know it”.

Thus Suarez-Kurtz builds his arguments through a conjunction of ideas about the history of the country, ‘mixed blood’, science and nation building, which are articulated with recent knowledge from the field of pharmacogenomics. In the term of Sommer (2010), we may say that Suarez-Kurtz recounts a ‘biohistory’ of the Brazilian genome, which in his perspective is seen as patrimony and ‘archive of history’ that must be taken in consideration when setting the priorities for research on the interface of human biological diversity and pharmacology. Such narratives have been extensively analyzed in the literature of anthropology, sociology and that of STS scholars, stretch outward from the individual level, passing through social groups, to reach the level of nations (see, for instance, Rabinow, 2002; Hinterberger, 2012). When Suarez-Kurtz says “It is most likely that this [Brazilian pharmacogenomic profile] applies to other admixed populations of the Americas”, he takes the Brazilian context as representative of a “Latin American imagined (pharmaco)genetic community”, (see Simpson, 2000) where the question of ‘mixed race’ would be regarded in a fundamentally different way than in regimes where the production of pharmacogenomic knowledge is based on membership in discrete ethno-racial groups. Hence, Suarez-Kurtz, in his own words, suggests that scientific knowledge should not lose sight of its roots when immersed in the complex and controversial realities of particular historical situations. In this sense, the significance of the pharmacogenomic research in Brazil is that it presents, at least in part, some elements of a counter discourse as it both makes use of and simultaneously challenges the globalization of (pharmaco)genes, bodies, ethno-racial categories and technology derived from particular contexts.^13^

Nevertheless, at the same time that the perspective espoused by Suarez-Kurtz on the matter of race and the pharmacogenomics of warfarin is far removed from some major lines of reasoning on the topic in the United States, there is some comparability in the way that its central message makes use of an emphasis on the individual. Thus, at the same time that the perspective of the Brazilian geneticists presents marked differences from the ones prevailing in the United States, it also paradoxically parallels some aspects, as it rests on and draws its force from recognizing the need for individualized treatments. The fact that this very prominent American discourse, that has been central to the pursuit of personalized medicine within pharmacogenomics, is also highlighted in the Brazilian context provides further evidence of how there is an ‘incorporation’ and not simply or only a rejection of specific aspects of US–European pharmacogenetic research.

Throughout this article, we have tried to show that pharmacogenomics in Brazil must be understood as scientific practice deeply embedded in the values and history of the society where it is produced. In this sense, there are significant differences but also similarities with arguments recently put forward by scholars who have analyzed the interface between ethnoracial categories and pharmacogenomics. If one of Fullwiley’s (2007) aims is to understand “how do scientists today use race as a biogenetic entity” (p. 2), our aim in this work has been not to understand race is ‘used’ as a biogenetic entity, but how do [Brazilian] scientists today simultaneously incorporate and reject race as a biogenetic entity. In the same way that Bliss (2011) points out that the scientists she interviewed “practice a reflexive form of biosociality in which they actively work to refashion ideas based on the kind of biosocial future they want for society” (p. 8), from the pharmacogenomic algorithms presented by Suarez-Kurtz a biosociality emerges that is deeply embedded in a positive discourse about miscegenation. In terms of health, the ‘biosocial future’ that pharmacogenomics pictures for Brazil is one where the biological characteristics of individuals, and not those of ‘race’, should be emphasized in the routines of drug administration, even as the individuality at stake must be read through the increasingly ubiquitous promise of personal medicine and highly particular histories and politics of race, color and national identity.

## Footnotes

1 For exceptions, see Caulfield *et al* (2009) and Urban (2010).

2 See Kent and Santos (2014), Maio and Santos (2010), Santos and Maio (2005) and Santos *et al* (2009, 2014a) for critical analyses on the incorporation of arguments derived from human population genomic research into the debate on race and affirmative action in Brazil.

3 Interviews with Suarez-Kurtz were carried out by Silva and Santos on 25 November 2010 and 5 April 2011 in his office at the National Cancer Institute (INCA) in Rio de Janeiro. The interviews were recorded and later transcribed for analysis.

4 In our interviews, Suarez-Kurtz affirmed that in the first research he did on the interface of race and pharmacogenomics was the one whose results were reported in Reis *et al* (2003). He referred critically to this study, because at the time he used the expressions “Afro-derived” and “Euro-derived” and “having interethnic admixture” that he ceased to use in favor of a focus more aligned with Pena’s theoretical approach.

5 Although the interplay between genes, geography and human variability patterns might not be regarded as a racialized approach by human geneticists, it tends to be associated with ‘race’ in the eyes of the non-experts. Concerning the uses of genetic ancestry informative markers, geneticists often argue that they comprise just a tiny, often non-codifying, portion of the human genome, thus not providing support to a perspective of race that emphasizes deep and consistent biological differences across racial groups. Notwithstanding, there are instances, as in the case of companies that market genetic ancestry tests, often aimed at specific ethnic minority groups such as the African-American, that end up propagating notions that associate genetics with racial belonging (Bolnick *et al*, 2007; Bolnick, 2008; Sommer, 2010).

6 See Mozersky and Gibbon (2014) for an account of how there is a similar questioning of the translation of risk variables in standardizing BRCA genetic testing for the Brazilian population and also Gibbon (2013).

7 One aspect that deserves emphasis is that over the last decade policies have been implemented especially for ethnic minorities, such as the “National Policy for Indigenous Health Care” of 2004, and the “National Policy for Health Care of the Black Population” of 2007. These policies do not relate specifically to the field of pharmacogenomics. In Brazil, pharmaceutical assistance is part of the national health system (Oliveira *et al*, 2007; Petryna, 2009, pp. 146–153). In recent years, several authors have discussed how the premise of universal access to health within the SUS system has led to a process known as judicialization of the right to health as patient litigants sue the government so that they can receive treatment (Pepe *et al*, 2010; Biehl and Petryna, 2013).

8 As exemplified by Hinterberger’s work in Canada and M’charek’s work in the Netherlands (Hinterberger, 2012; M’charek, 2013), respectively, there has been a growing interest in examining the multiple manifestations of ‘race’ in genome sciences in different national contexts, thus shedding light on how different historical and socio-political contexts might influence the incorporation and rejection of race. Along with our Brazilian case study, this recent body of research enriches the spectrum of critiques in this area which have been, until recently, to a large extent based on US perspectives on race and science.

9 Following a presentation of this article in a colloquium at the Max Planck Institute for the History of Science, in Berlin, Veronika Lipphard made an insightful comment that link some of the ideas we discuss here to key events in the history of physical anthropology. For her, the “racialize to deracialize” approach of the Brazilian pharmacogeneticists is echoed in Franz Boas’ research in the 1910s on the changing body forms of descendants of immigrants to the United States. Boas (1912) used long-established anthropometric techniques to show that facial and cranial characteristics (dolichocephaly, brachicephaly and so on) of the descendants born in the United States could be different from their parents. Thus, Boas deployed classical physical anthropology procedures to undermine notions of racial fixity that, to a large extent, were built by physical anthropologists in the second half of the nineteenth century (see also Stocking, 1974).

10 Although there is a sizable literature on the clinical and genetic aspects of warfarin dosing, including the proposition of algorithms, we are not aware of any study that has investigated from a social science perspective the actual practices of warfarin dosing by medical personal in hospitals and other health provisioning contexts. Anthropological–ethnographic research could potentially illuminate how, in practice, health professionals perceive and use the dosing algorithms.

11 The examples of warfarin dosing algorithms we have discussed so far reinforce the perspective of genotype-guided dosing of warfarin. It should be mentioned, however, that there are recent publications that argue that genotyping does not improve clinical procedures (see, for instance, Kimmel *et al*, 2013).

12 It is perhaps important to note also the parallels between the movement of ‘anthropophagy’ that Tarsila do Amaral was part of in the early part of the twentieth century and that sought (in a similar way to the Brazilian geneticists described in this article) to incorporate and reject European painting and artistic styles and forms (Gonçalves, 2012). In this sense the choice of images by the Brazilian pharmacogeneticists is telling.

13 In this article, which addresses the particular case of warfarin, we are not implying that in other areas of genetic research human geneticists, pharmacologists and other scientists in the United States do not take into account the issue of admixture. Even a brief search in broadly used bibliographic databases, like PubMed-line, show that admixture is an important topic of research in human genetics in the United States. However, this does not seem to be the case in the literature on warfarin dosing algorithms.
